# A computational modelling study of excitation of neuronal cells with triboelectric nanogenerators

**DOI:** 10.1038/s41598-022-17050-0

**Published:** 2022-08-04

**Authors:** Razieh Nazari-Vanani, Raheleh Mohammadpour, Elham Asadian, Hashem Rafii-Tabar, Pezhman Sasanpour

**Affiliations:** 1 Department of Medical Physics and Biomedical Engineering, School of Medicine, Shahid Beheshti University of Medical Sciences, Tehran, Iran; 2 Institute for Nanoscience and Nanotechnology (INST), Sharif University of Technology, Tehran, Iran; 3 The Physics Branch of Iran Academy of Sciences, Tehran, Iran; 4 School of Nanoscience, Institute for Research in Fundamental Sciences (IPM), P. O. Box 19395-5531, Tehran, Iran

**Keywords:** Computational science, Neurological disorders, Neurological models, Biomedical engineering

## Abstract

Neurological disorders and nerve injuries, such as spinal cord injury, stroke, and multiple sclerosis can result in the loss of muscle function. Electrical stimulation of the neuronal cells is the currently available clinical treatment in this regard. As an effective energy harvester, the triboelectric nanogenerators (TENG) can be used for self-powered neural/muscle stimulations because the output of the TENG provides stimulation pulses for nerves. In the present study, using a computational modelling approach, the effect of surface micropatterns on the electric field distribution, induced voltage and capacitance of the TENG structures have been investigated. By incorporating the effect of the TENG inside the mathematical model of neuron’s electrical behavior (cable equation with Hodgkin-Huxley model), its impact on the electrical behavior of the neurons has been studied. The results show that the TENG operates differently with various surface modifications. The performance of the TENG in excitation of neurons depends on the contact and release speed of its electrodes accordingly.

## Introduction

Humans are surrounded by various types of energy sources in the environment including solar, thermal, mechanical, chemical and biological energies^1^. In addition, the human body itself is a favorable source of energy as it is semi-permanent and easily available^2^. Furthermore, development of sustainable energy sources is an inevitable demand due to the emergence of portable electronic devices and sensor networks^3^. Among various types of energy sources, mechanical energy has attracted considerable attention owing to its universal availability in the natural environment, human body as well as living activities^4^. Therefore, research has been conducted to develop integrated systems called *Nanoenergy,* exploiting micro and nanostructures capable of easily harvesting the energy from the environment and operate continuously, independently and effectively^5–7^. Energy-harvesting techniques form possible solution to the power-supply issue of portable electronic devices, healthcare devices, and wireless sensors^8^ which are currently supplied by batteries. Application of usual batteries is getting impractical and unfavorable, mainly because of their limited lifetime, maintenance difficulties, and environmental hazards considering the leakage of the chemicals^9,10^. Beside the traditional technologies for mechanical energy harvesting to provide the energy for electronic devices, the triboelectric nanogenerators (TENGs) have received close attention in recent years due to their strong potential for use in self-powered systems^11^. The TENGs are widely used to harvest and convert mechanical energy into electrical energy in different applications^12^. The working principle of the TENG is based on contact triboelectrification along with electrostatic induction effects^13^. As an old familiar phenomenon, the triboelectric effect occurs between two materials with different triboelectric affinities and leads to the transfer of charges and an increase in the tribo-potential^14,15^. When two materials are in contact and then separated, the alternating potential will drive electrons into the external electric circuit causing them to move back and forth^16^.

The TENGs have been widely used^17,18^ in various biomedical applications, such as cancer therapy^19^, pacemaker^20^, glucose monitoring^21^, sensors^22,23^, ion detection^24^, as well as in devices for muscle stimulation^25^ due to their considerable output power, low weight, easy fabrication process with low cost, environmental compatibility, abundant available selection of materials, universal availability, and simple structure.

The measurement of electrophysiological signals from muscles or neural tissues is of great importance in the diagnosis of many neuronal dysfunctions while the electrical stimulation of the spinal cord and neurons can be utilized for the treatment of certain diseases^26,27^. The damage to human nervous system during stroke or spinal cord injuries would result in the weakness of the muscles or atrophy and might deteriorate to paralysis^28,29^. The lack of neuronal innervation due to neurological damage, promotes muscle inability to produce the voluntary forces needed to create the movement of the joints^30^. Hence, numerous scientific investigations have focused on the devices, and strategies to assist the body to restore muscle atrophy, recover muscle movement and function after injury or surgery^30,31^.

In this regard, the TENGs can simultaneously serve as both a waveform generator and a power source for electrical stimulation of muscles^29^. The output of the TENG can be directly used for stimulation of nerves and muscles^29,32^. Inside the human body, electrical signals carry stimulus information and regulate neuronal activities. Neurological disorders and nerve injuries can lead to the loss of muscle function with increasing muscle atrophy as initial symptoms, which ultimately might culminate in the paralysis. Electrical muscle stimulations have been applied as a powerful tool to treat neurological disorders, prevent and restore muscle atrophy, and recover muscle movements^31^.

In the past few years, electrical currents generated from the TENGs have been successfully used in various studies for the electrical stimulation of the cells^33,34^, nerves^35^, and the brain^36^. On the cell level, a TENG-driven electric stimulation system has been designed for promoting cellular proliferation of the L929 cells which demonstrates the effectiveness of the TENG and its safe operational conditions in biomedical stimulation^37^. The results demonstrate that the TENG-based stimulation regulates the cell proliferation and migration of the fibroblast cells^37^. Li et. al.^33^ fabricated a TENG that produced stable pulsed current output to stimulate the rejuvenation of aged mesenchymal stromal cells. The results indicated that the triboelectric stimulation improved the proliferation of aged bone marrow mesenchymal stromal cells and increased their pluripotency and differentiation capacity.

On the peripheral nerve level, Zhang et al. first demonstrated the direct TENG stimulation with a peak output voltage of 265 V and current density of 18.3 μA/cm^2^ which was also successfully applied to stimulate a frog’s sciatic nerve^38^. In another study conducted by Lee et al., a stacked TENG with the configuration of patterned polydimethylsiloxane, polyethylene terephthalate film and Cu electrode was exploited as a potential power source for neural stimulation and proved to generate the output voltage and short circuit current of 160 V and 6.7 μA, respectively. Operating the device could directly stimulate the rat’s sciatic nerve, while controlling the muscle contraction and monitor the muscle signals^39^. In another study, Lee et. al.^40^ developed a novel water/air-hybrid TENG for peripheral nerve stimulation. Yao and coworkers^41^ presented a TENG-powered implanted nerve stimulation system on the rat’s vagus nerve for reduction in food intake and achieved the weight control. The nerve stimulation system was battery-free and spontaneously responsive to the stomach movements. The strategy was successfully applied to the rat models. Within 100 days, the average body weight was controlled which was 38% less than the control group. On the brain level, Dai et al.^42^ demonstrated direct TENG stimulation of the rat’s somatosensory cortex and motor cortex. The device, which was connected to the mouse’s brain at primary somatosensory barrel cortex, could mimic the mouse perception and drove the mouse activities.

As mentioned previously, the high-performance TENGs could be achieved through an optimized device structure, proper selection of functional materials in the triboelectric series and surface modifications to enhance the contact area^17^.

Simulation methods are powerful techniques offering deep insight into the working mechanism of devices, material selection, exploring new features, operating conditions and analyzing the output performance of the TENG systems^43^. It seems necessary to study the mechanism in each application, while calculating the values for various conditions through laboratory tests is usually a time-consuming, expensive and lengthy task^44^. Moreover, simulation results could accelerate the design of novel systems. To this end, computational modelling and simulation have been employed to determine the appropriate TENG structure and materials, and avoiding designs which would decrease the output performance^3^.

In this paper, based on a multiphysics computational modelling approach, we have studied the performance of the TENG structure for excitation of neuronal cells. The effect of surface structures and morphology of different substrates, as well as the velocity of striking, have been studied in the evoked action potentials of the neurons accordingly. Using the finite element method (FEM) in the COMSOL environment and through finding the electric field distribution for various structural design parameters, the values of the generated voltage and capacitance were initially calculated for a TENG in a contact-separation mode in each relative position of its electrodes. The constructed model could be exploited as an effective design tool not only for the prediction of the response of a contact-separation mode TENG, but also for the selection of optimum values of the structural parameters such as width, height and the distance between the micro-structures^11^. Then, the intrinsic output characteristics of the open-circuit voltage (V_oc_), and the previously calculated inherent capacitance of the contact-separation mode are coupled to the cable equation with the Hodgkin-Huxley (H–H) model to represent the electrophysiological behavior of the neuron in response to the TENG structure. To the best of our knowledge, this is the very first computational study focused on representing the effect of the TENG structure on the electrophysiological behavior of the neurons.

## Modeling approach

**Figure 1:**
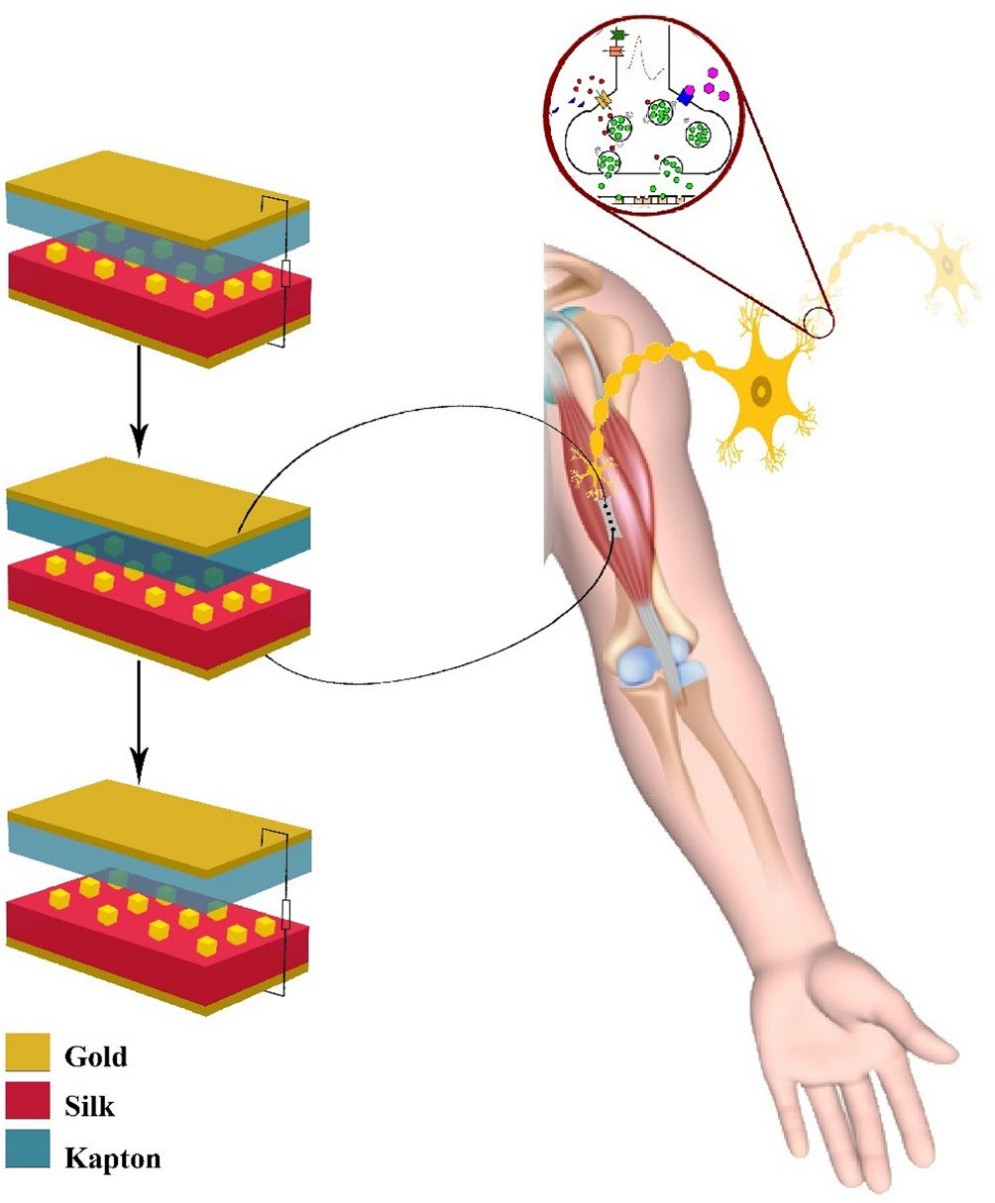
Schematic diagram of the operating TENG and electrical stimulation of the neuron.

The close contact between the TENG and neuron structure is schematically illustrated in Fig. 1 for which, the values of the output voltage and capacitance are calculated at various locations of the electrodes by solving the Poisson equation. The response of the neuron to the excitation from the TENG is calculated based on the developed model including the cable equation (with the H–H model) coupled with the TENG model.

### TENG operation modelling

**Figure 2:**
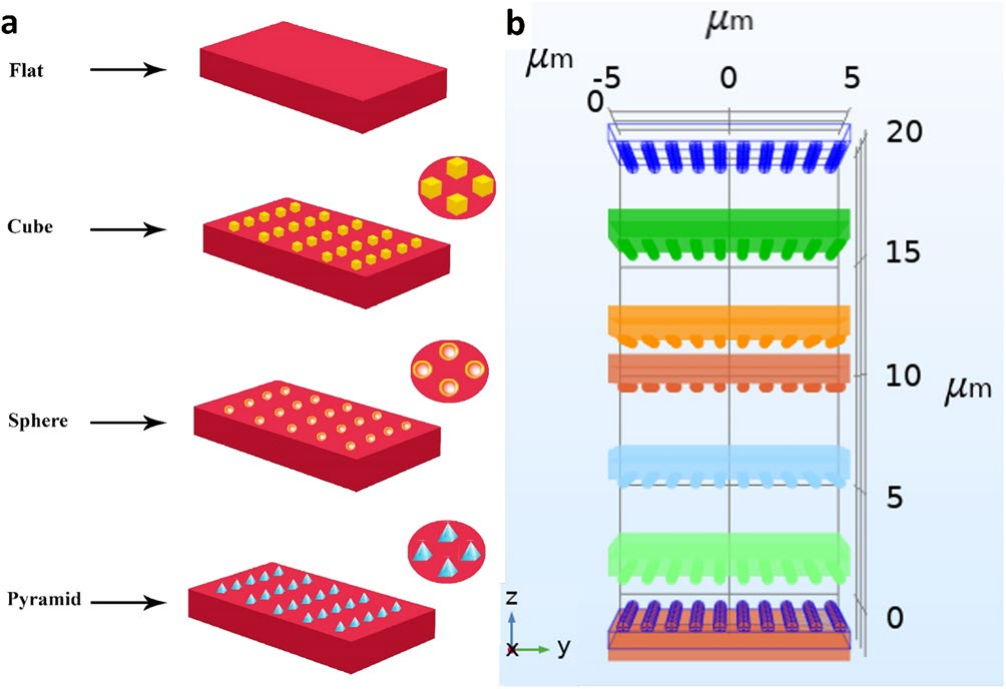
(**a**) Geometrical representation of the TENG electrodes with different surface modifications. (**b**) Representation of the computational model in the COMSOL environment for calculation of capacitance and charge for different distances between the TENG’s electrodes.

Considering the influence of the morphology of substrate’s surface material, and its geometry on the local electric field and surface charge density, a 3D model incorporating various types and geometrical parameters was developed. The Poisson equation was solved for the structure for distinct distances between the two substrates. After finding the electric field distribution, the capacitance for each structure was calculated by Eq. (1). The model considered not only the effect of surface roughness, but also the variation in the distance between the two substrates on the output performance. Considering the size and the frequency range, the AC/DC Module with the electrostatic physics was considered. Figure 2a shows the geometrical model developed in the COMSOL environment for various distances (Fig. 2b) between the substrates with different electrode surface modifications. To this end, a TENG with vertical contact-separation mode was constructed based on silk and polyimide (Kapton) as triboelectric materials and two gold (Au) layers were also considered as metal electrodes. Silk and polyimide were selected since they are located in almost two ends of the triboelectric series (43). The parameters used for the TENG in the model are given in Table 1.

**Table 1:** Parameters used in the calculation of TENG’s output characteristics.

Parameters	Values and units
Surface area of layers	15 × 10^–5^ m^2^
Thickness of dielectrics layer	7 × 10^–7^ m
Thickness of electrodes	5 × 10^–7^ m
Surface charge density	5 × 10^–5^ C m^−2^
Relative dielectric constant of silk layer	2.6
Relative dielectric constant of Kapton layer	3.4

At the outer surfaces of simulation box, zero charge was considered as the boundary condition while surface charge density was considered for the inner side of electrodes of the TENG.

The electric potential difference between the two substrates (open circuit output, V_oc_) was derived and the capacitance was calculated using

1 $${\text{C}} = \frac{Q}{{V_{OC} }}$$

where V_OC_ is the open circuit output voltage and *Q* denotes the triboelectric charge.

### Neuron modelling

#### Cable equation

The propagation of action potential in an axon is an electrophysical process that has been studied using the cable theory coupled with the Hodgkin-Huxley (H–H) model involving different ionic currents^45^. In this regard, the cable Eq. (2) was used to find the behavior of the membrane in response to the external stimuli which is derived on the basis of the core-conductor model^46^

2 $$C_{m} \frac{{\partial V_{m} \left( {z,t} \right)}}{\partial t} = \frac{1}{{\left( {2\pi a} \right)\left( {r_{i} + { }r_{e} } \right)}}\frac{{\partial^{2} V_{m} \left( {z,t} \right)}}{{\partial z^{2} }} + I_{S } - I_{ion} \left( {z,t} \right)$$

where *a* is the radius of the cylinder (axon), V_m_ is the transmembrane potential, I_s_ is the transmembrane stimulation current, C_m_ is the membrane’s capacitance per unit area, r_e_ and r_i_ are the extracellular and intracellular axial resistances per unit length respectively, and I_ion_ is the sum of different ionic current components (Na, K and other components).

#### Hodgkin-Huxley model

The H–H model was developed in 1952 as a result of Hodgkin’s and Huxley’s extensive studies of the giant axon of the squid^47^. It describes how the action potential is initiated and how it propagates in a neuron^48^. The ionic currents through the membrane can be calculated by Eq. (3).

3 $$I_{ion} = {\text{g}}_{{\text{K}}} { }\left( {{\text{V}}_{{\text{m}}} - {\text{E}}_{{\text{K}}} } \right) + {\text{g}}_{{{\text{Na}}}} { }\left( {{\text{V}}_{{\text{m}}} - {\text{E}}_{{{\text{Na}}}} } \right) + {\text{g}}_{{\text{L}}} { }\left( {{\text{V}}_{{\text{m}}} - {\text{E}}_{{\text{L}}} } \right)$$

where g_L_ is the leak conductance, g_Na_ is the sodium conductance, g_K_ is the potassium conductance, V_m_ is the transmembrane potential, E_L_ is the Nernst potential for the leak conductance, E_Na_ is the Nernst potential for the sodium channel, and E_K_ is the Nernst potential for the potassium channel.

The values of the conductances, g_Na_ and g_K,_ are formulated based on the H–H experiment on the giant squid axon^46,49^ and g_L_ is considered fixed, and

4 $${\text{g}}_{{\text{K}}} = {\overline{\text{g}}}_{{\text{K}}} {\text{n}}^{4} { }$$

5 $${\text{g}}_{{{\text{Na}}}} = {\overline{\text{g}}}_{{{\text{Na}}}} {\text{m}}^{3} {\text{h}}$$

where n, h, and m are gating variables, which are constrained between 0 and 1, and represent the probability for opening or closing of a channel. The terms with bar represent the maximum conductance of each channel.

Table 2 shows the parameters used in the cable equation and the H–H model.

**Table 2:** The parameters used in the model.

Symbol	Model parameters	Value
E_Na_	Sodium reversal potential	52.4 mV
E_K_	Potassium reversal potential	72.1 mV
E_L_	Leakage reversal potential	49.2 mV
g_Na_	Sodium conductance	120.0 mS/cm^2^
g_K_	Potassium conductance	36 mS/cm^2^
g_L_	Leakage conductance	0.3 mS/cm^2^
C	Membrane capacitance	1.0 μF/cm^2^
a	Axon radius	0.001 cm
V_r_	Resting potential	60 mV
R_i_	Resistivity of intracellular	35 Ω.cm
R_e_	Resistivity of extracellular	20 Ω.cm

#### Extended cable equation

In order to incorporate the effect of the TENG’s presence on the neuron’s electrophysiological behavior and the propagation of the action potential, its influence was introduced in the cable equation as an external current (Eq. 6).

6 $$\begin{aligned} C_{m} \frac{{\partial {\text{V}}_{m} }}{{\partial {\text{t}}}} = & \frac{1}{{\left( {2\pi a} \right)\left( {r_{i} + { }r_{e} } \right)}}\frac{{\partial^{2} V_{m} \left( {z,t} \right)}}{{\partial z^{2} }} + I_{ion} \left( {{\text{HH}}} \right) - \frac{{{\text{dC}}\left( {\text{x}} \right)}}{{{\text{dx}}}}\frac{{{\text{dx}}}}{{{\text{dt}}}}\left( {{\text{V}}_{oc} \left( {\text{x}} \right) - {\text{V}}_{m} } \right) \\ & - {\text{C}}\left( {\text{x}} \right)\frac{{{\text{dV}}_{oc} \left( x \right)}}{{{\text{dx}}}}\frac{{{\text{dx}}}}{{{\text{dt}}}} + {\text{C}}\left( {\text{x}} \right)\frac{{\partial {\text{V}}_{m} }}{{\partial {\text{t}}}} \\ \end{aligned}$$

In order to numerically solve the equation, all the derivatives were replaced with differences (Eq. 7) and finally Eq. (8) was derived accordingly.

7 $$\begin{aligned} C_{m} \frac{{V_{m}^{i + 1} - V_{m}^{i} }}{\Delta t} = & \frac{1}{{\left( {2\pi a} \right)\left( {r_{i} + { }r_{e} } \right)}}\frac{{\partial^{2} V_{m}^{i} \left( z \right)}}{{\partial z^{2} }} + g_{K}^{i} \left( {V_{m}^{i} - E_{k} } \right) + g_{Na}^{i} \left( {V_{m}^{i} - E_{Na} } \right) + g_{L}^{i} \left( {V_{m}^{i} - E_{L} } \right) \\ & - \frac{{{\text{C}}\left( {x + {\Delta x}} \right) - {\text{C}}\left( {\text{x}} \right)}}{{{\Delta x}}}\frac{{{\text{dx}}}}{{{\text{dt}}}}\left( {V_{oc} \left( x \right) - V_{m}^{i} } \right) - {\text{C}}\left( {\text{x}} \right)\frac{{V_{oc} \left( {x + {\Delta x}} \right) - V_{oc} \left( x \right)}}{{{\Delta x}}}\frac{{{\text{dx}}}}{{{\text{dt}}}} + {\text{C}}\left( {\text{x}} \right)\frac{{V_{m}^{i + 1} - V_{m}^{i} }}{{{\Delta t}}} \\ \end{aligned}$$

8 $$\begin{gathered} V_{m}^{i + 1} = V_{m}^{i} + \frac{\Delta t}{{C_{m} - C\left( x \right)}}\frac{1}{{\left( {2\pi a} \right)\left( {r_{i} + { }r_{e} } \right)}}\frac{{\partial^{2} V_{m}^{i} \left( z \right)}}{{\partial z^{2} }} + \frac{\Delta t}{{C_{m} - C\left( x \right)}} \left( {g_{K}^{i} \left( {V_{m}^{i} - E_{k} } \right) + g_{Na}^{i} \left( {V_{m}^{i} - E_{Na} } \right) + g_{L}^{i} \left( {V_{m}^{i} - E_{L} } \right)} \right) \hfill \\ - \frac{\Delta t}{{{\Delta x}}}\frac{{{\text{C}}\left( {x + {\Delta x}} \right) - {\text{C}}\left( {\text{x}} \right)}}{{C_{m} - C\left( x \right)}}\frac{{{\text{dx}}}}{{{\text{dt}}}}\left( {V_{oc} \left( x \right) - V_{m}^{i} } \right) - \frac{\Delta t}{{{\Delta x}}}\frac{{{\text{C}}\left( {\text{x}} \right)}}{{C_{m} - C\left( x \right)}}\left( {V_{oc} \left( {x + {\Delta x}} \right) - V_{oc} \left( x \right)} \right)\frac{{{\text{dx}}}}{{{\text{dt}}}} \hfill \\ \end{gathered}$$

where dx/dt represents the contact-release speed of the two the substrates and C denotes the capacitance between the TENG electrodes. Subscript *m* represents the membrane and superscript *i* denotes the *i*th time-step.

The complete set of equations, which describes the generation and propagation of the action potentials for any axon, was solved numerically using our own developed code in the MATLAB.

## Results

### TENG output specifications

As mentioned previously, a vertical contact-separation mode was considered for the TENG setup wherein the bottom electrode was fixed while the top electrode was free and could move up and down. Two Au electrodes were also considered as the electrical contacts with the tribo-material (i.e. silk and Kapton). In order to study the effect of surface roughness, the simulations were performed for the surfaces with four different geometrical arrangements; flat surface (without micropattern) as well as surface covered with cube, pyramid, and sphere-shaped micropatterns (Fig. 2a).

**Figure 3:**
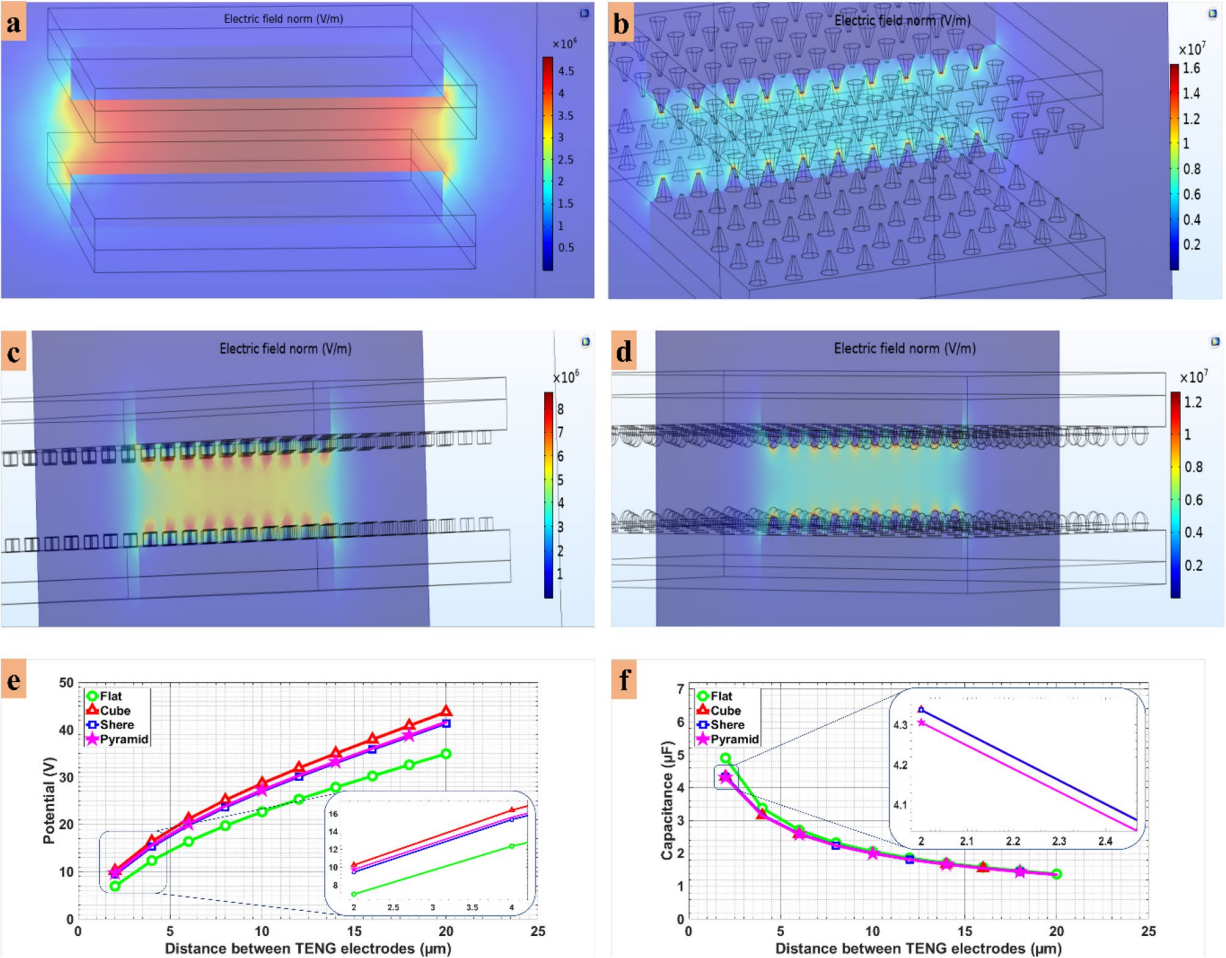
Electric field distribution between surfaces of the TENG for (**a**) flat shape surface and surfaces with (**b**) pyramidal, (**c**) cubic, (**d**) spherical shape micropatterns, (**e**) calculated open circuit voltage vs. distance, and (**f**) calculated capacitance between two the electrodes vs. distance.

Figures 3a–d illustrate the electric field distribution between the TENG electrodes with different micropatterns. Considering a simple model of two planar surface electrodes, it is expected that the electric field reaches its maximum value when the distance between the two substrates is at minimum. As can be clearly seen in Fig. 3e, the micropatterned structures exhibited a higher voltage output compared to the flat surface. Moreover, the output voltage for the cube-shaped TENG was higher than the two other micropatterns. The results demonstrated that the capacitance of the TENG decreased slightly with an increase in the distance between the two electrodes (Fig. 3f).

### Neuronal behavior in response to the TENG output

The intrinsic properties of the action potential propagation along the axon in presence and absence of the nanogenerator were calculated based on the developed equation and in combination with the results of the FEM for the TENG specifications.

**Figure 4:**
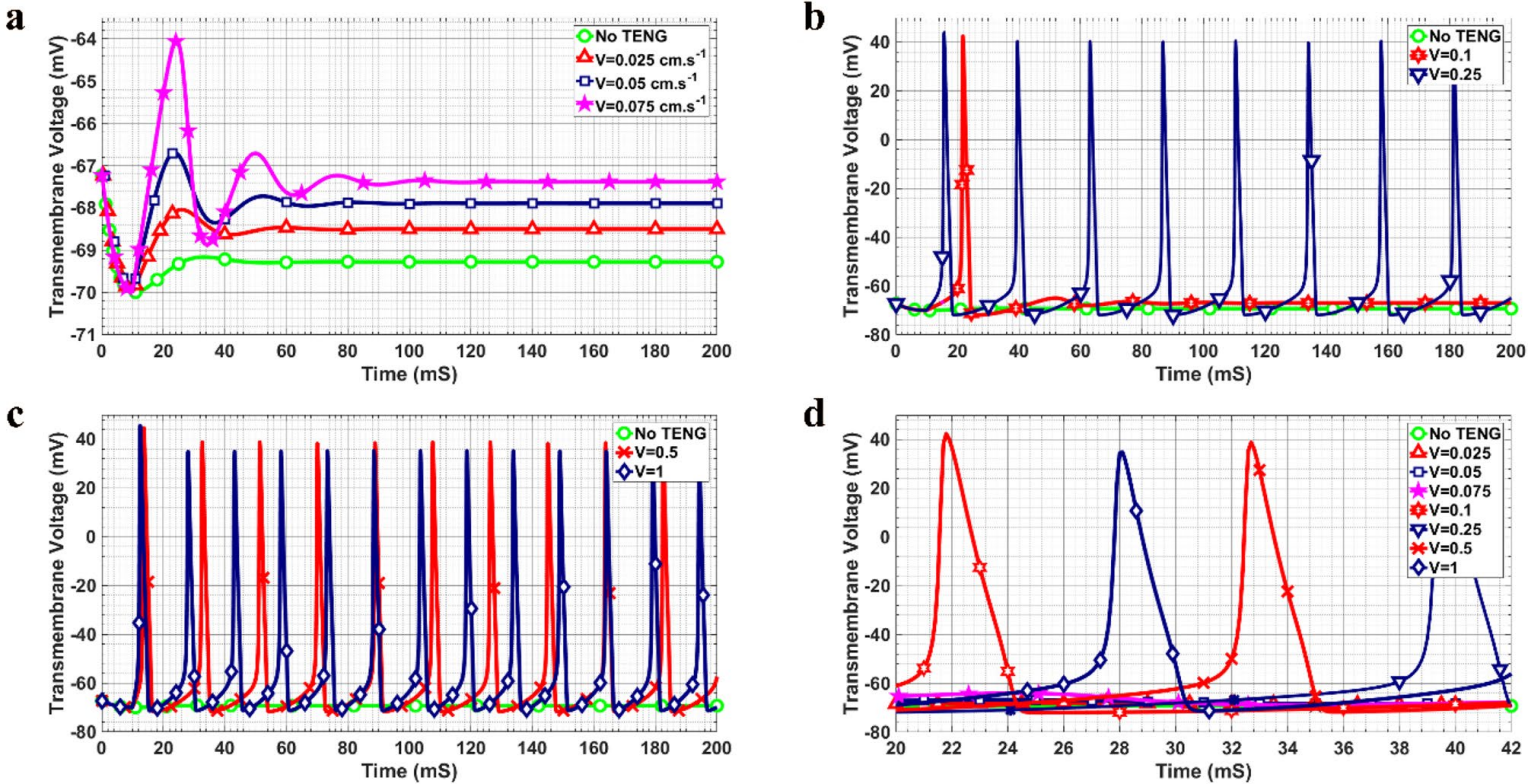
Transmembrane voltage of the resting neuron for different contact-release speeds of (**a**) 0.025–0.075 cm.s^−1^ and without the TENG, (**b**) 0.1–0.25 cm.s^−1^, (**c**) 0.5–1 cm.s^−1^, (**d**) results for the time duration between 20 and 42 ms.

Figures 4a–d, show the effect of different values of contact-release speed of the two substrates on the formation of the action potential in the axon of the neuron. Initially, the neuron was in the rest state while with the application of the TENG, it was excited. As depicted in Fig. 4a, for the speed between 0 and 0.075 cm.s^−1^, no action potential was evoked. In other words, in this speed range, the membrane voltage did experience some fluctuations. It should be noted that in this case, by increasing the speed of the contact-release process, a slight increase in the rest potential of the neuron was observed. By further increasing the contact-release speed to 0.1 cm.s^−1^ (Fig. 4b), there was a single evoked action potential for the resting neuron. For the speed in the range of 0.25 to 0.5 cm.s^−1^, the resting neuron started to fire action potentials (Figs. 4b and 4c). Figure 4d compares the results of the TENG for all the speeds in the time duration between 20 and 42 ms. Moreover, the transition from hyperpolarization to the rest state occurred more rapidly by increasing the speed of the contact-release process.

**Figure 5:**
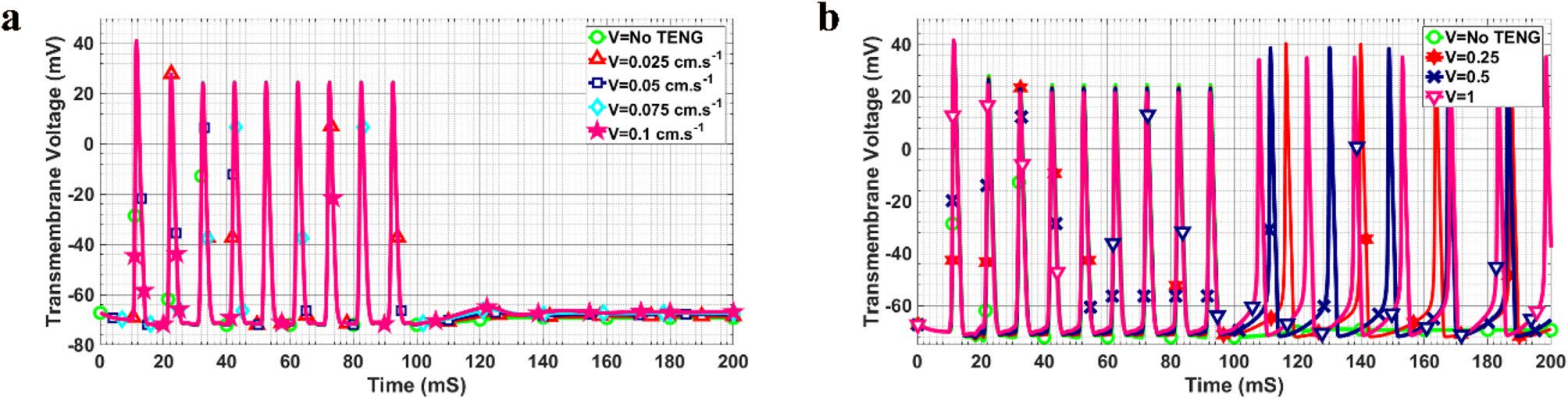
Transmembrane voltage of the firing neuron for different contact-release speeds of (**a**) 0.025–0.1 cm.s^−1^ and without TENG, (**b**) 0.25–1 cm.s^−1^.

In order to find the effect of the TENG on the firing neuron, we considered a mathematical model of the neuron which fired between 0 to 100 ms and the effect of the TENG was considered on its performance. Figures 5a-b show the transmembrane voltage of the firing neuron in presence of the TENG for different values of the contact-release speed in the time duration of 0 and 200 ms. It was observed that for all of the speeds considered, during the period that the neuron was in the firing state (0 to 100 ms), the TENG imposed no tangible effect on the action potentials. When the neuron stopped firing (100 ms to 200 ms), the effect of the TENG on the neuron was similar to the resting neuron (Figs. 4a–c). Overall, for small speed values, there was no evoked action potential and just an elevation in the transmembrane potential occurred (Fig. 5a) while for higher speed values, the neuron started to fire (Fig. 5b). In addition, as can be clearly seen in Fig. 5b, there was a slight reduction observed in the amplitude of the action potentials with increasing speed.

### Surface roughness effect

**Figure 6:**
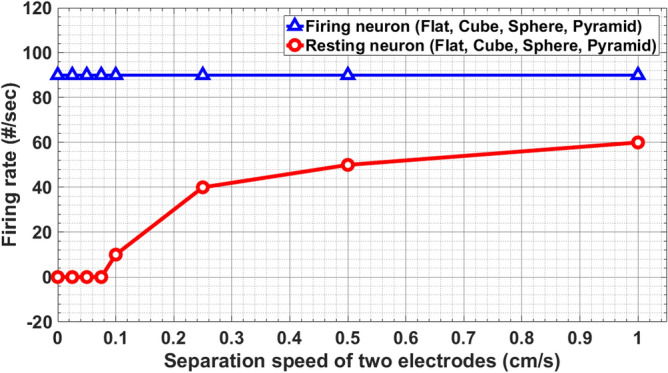
Variations of the firing rate with speed for the resting and firing states in response to the TENG for different electrode structures.

In order to compare the efficiency of the flat TENG with those of the micropatterned in exciting the neurons, the role of various morphologies was considered in the computational model accordingly. The results showed the same trend with slight changes. For the resting neuron, as can be seen in Figs. 4a–c, the action potentials were evoked with different time behavior. Figure 6 shows the variations of the firing rate with the contact-release speed for the resting and firing neuron in response to the TENGs with different electrode structures. As can be observed from the plot, the speed of the contact-separation process had a direct impact on the firing rate so that it increased with the rising speed, while the surface morphology of electrodes did not affect the firing rate.

**Figure 7:**
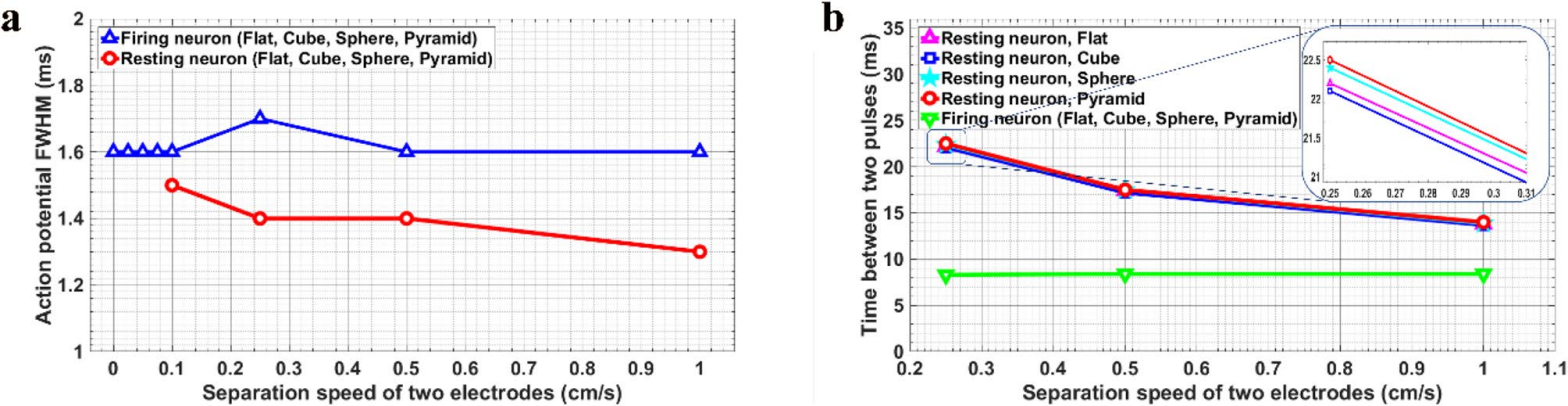
(**a**) FWHM of action potential during propagation. (**b**) Time delay between two consecutive pulses.

Based on the importance of pulse broadening, the values of full width at half maximum (FWHM) of the generated action potentials were compared for different contact-release speeds in presence of the TENG. Figure 7a shows the results of the variations of the calculated FWHM with the contact-release speed for different electrode structures. The results show a slight decrease in the FWHM with an increase in the speed for both firing and resting neuron. For all of the cases, peak broadening was not occurred.

Figure 7b shows the time delay between two consecutive evoked action potentials for different contact-release speeds. The results indicate that by increasing the contact-release speed, the time delay between two successive pulses had decreased. The same behavior was observed for all types of electrodes with a negligible difference for various surface microstructures.

## Discussion

In the present paper, we have studied the influence of the TENG structure on the excitation of neuronal cells. The effect of surface roughness as well as the contact-release speed of the two substrates on the evoked action potential of the neurons have been studied. First, by finding the electric field distribution of the TENG’s electrodes with different surface structures, the values of the generated voltage and capacitance of the structures in each relative position of the electrodes were calculated. As shown in Figs. 3e–f, the structures covered with micropatterns exhibited higher voltage output and lower capacitance values compared with the flat one. Moreover, the output voltage for the cube-shaped TENG was higher than the two other micropatterns, which confirms that the micropatterns increase both the surface charge and the surface area. The results are in good agreement with previous studies^50^. Muthu and Yinhu have discussed the effect of surface patterns on the TENG output voltage and capacitance^11,51^. Zhang et al. tried to use surface micropattern structures to increase the electric output which indicated that the films with pyramid arrays are optimal shapes with appropriate performance^52^. Compared with the flat films, pyramid arrays on the surface enhanced the voltage and the current by 100% and 157%. To confirm the influence of patterning on the TENG device, a simulation study has been conducted by Muthu et al.^51^. The simulation results showed that the output voltage of the TENG with linear patterns on the surface was significantly improved compared with flat, circle, and X pattern thin films. In addition to the effect of the surface area, Yinhu et al. group studied the effect of surface microstructures on the capacitance in TENG^11^. They found that introducing microstructures decreased the capacitance of the structure. In our study, the effect of induced voltage and the capacitance in the contact-separation mode was coupled to the cable equation within the Hodgkin-Huxley model to represent the electrophysiological behavior of the neuron in response to the TENG movement. The results (Figs. 4a–c and 5a–b) show that by increasing the contact-release speed, the neuron would start to fire. Small values of the speed resulted in no action potential or single action potential while by increasing the contact-release speed the neuron started to fire. This finding could be associated with the fact that at a lower speed, the ion channels were not active because of small level of induced voltage on the cell membrane. Furthermore, in the case of no action potential, the resting potential of the neuron was slightly elevated.

The firing rate is known as an electrophysiological characterization of neuromuscular disorders^53^. Parkinson’s disease reveal hyperactive neurons in the subthalamic nucleus that have increased firing rates and bursting activity compared with controls^54^. Figure 6 explains the firing rate dependency on the contact-release speed of the two substrates in which, the firing rate increased with the speed.

Figures 7a–b show the results of the calculated FWHM and the time delay between two consecutive evoked action potentials *vs*. the contact-release speed for different structures. The results display a slight decrease in the FWHM with an increase in the speed for both firing and resting neuron. The variation in FWHM does not change for different microstructures on the surface of electrodes. By increasing the contact-release speed, the time between two consecutive pulses becomes shorter and the peaks become narrower. This behavior is consistent with the results of the firing rate. For the firing neuron, the presence of the TENG had no obvious effect on the time between two consecutive pulses.

The main focus of this study was on the vertical contact-separation mode as an ideal model of the TENG, while other modes of operation could be considered for further studies.

## Conclusion

Based on the importance of the influence of the TENG on stimulation of the excitable cells, we have computationally modelled the effect of the TENG on the electrophysiological behavior of the neurons. The effect of the electrodes with different surface modifications on the induced voltage of the TENG and its capacitance in different positions was calculated. The effect of presence of the TENG on the neuron activity was introduced in the cable equation within the H–H model. The results show that the microstructured electrodes resulted in different output voltages and capacitance of the TENG. Regarding the stimulation of neurons with the TENG structures, the results indicate that the contact-release speed of the TENG had a direct influence on the evoking and propagation of action potentials in neurons.

## Data Availability

Derived data supporting the findings of this study are available from the corresponding author on request.
